# Microbial Hydrocarbon Degradation in Guaymas Basin—Exploring the Roles and Potential Interactions of Fungi and Sulfate-Reducing Bacteria

**DOI:** 10.3389/fmicb.2022.831828

**Published:** 2022-03-09

**Authors:** Virginia P. Edgcomb, Andreas P. Teske, Paraskevi Mara

**Affiliations:** ^1^Woods Hole Oceanographic Institution, Woods Hole, MA, United States; ^2^Department of Earth, Marine and Environmental Sciences, University of North Carolina at Chapel Hill, Chapel Hill, NC, United States

**Keywords:** hydrocarbon, fungi, sulfate-reducing bacteria, microbial interaction, Guaymas Basin

## Abstract

Hydrocarbons are degraded by specialized types of bacteria, archaea, and fungi. Their occurrence in marine hydrocarbon seeps and sediments prompted a study of their role and their potential interactions, using the hydrocarbon-rich hydrothermal sediments of Guaymas Basin in the Gulf of California as a model system. This sedimented vent site is characterized by localized hydrothermal circulation that introduces seawater sulfate into methane- and hydrocarbon-rich sediments, and thus selects for diverse hydrocarbon-degrading communities of which methane, alkane- and aromatics-oxidizing sulfate-reducing bacteria and archaea have been especially well-studied. Current molecular and cultivation surveys are detecting diverse fungi in Guaymas Basin hydrothermal sediments, and draw attention to possible fungal-bacterial interactions. In this Hypothesis and Theory article, we report on background, recent results and outcomes, and underlying hypotheses that guide current experiments on this topic in the Edgcomb and Teske labs in 2021, and that we will revisit during our ongoing investigations of bacterial, archaeal, and fungal communities in the deep sedimentary subsurface of Guaymas Basin.

## Introduction

**FIGURE 1:**
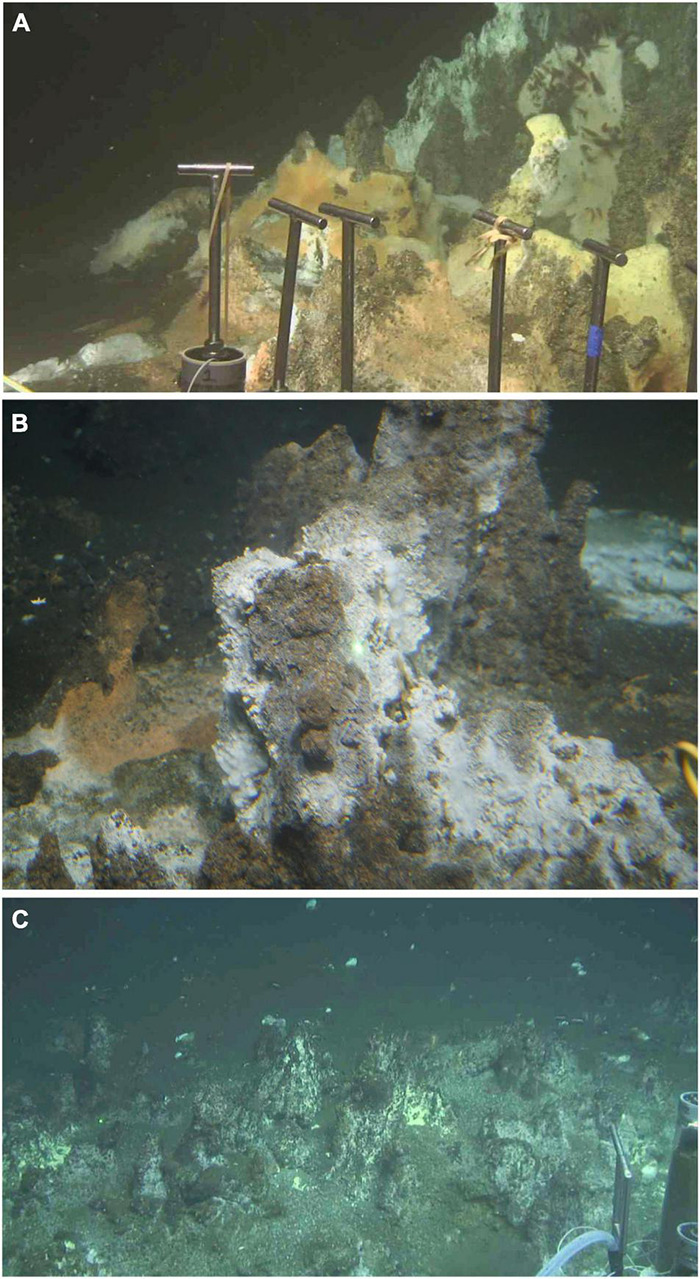
Hydrocarbon-rich hydrothermal sites in Guaymas Basin. **(A)** Hydrocarbon-soaked concretions at the base of the “Rebecca’s Roost” hydrothermal edifice (27°N00.70′/111°W24.23′). Nearby hydrocarbon-rich sediments were sampled by Deep-sea submersible *Alvin* in December 2016, and analyzed for geochemical properties and microbial community composition (Alvin core 4870-16, temperatures 31–52°C; Song et al., 2021 and Teske et al., 2021a). **(B)** Oil-soaked hydrothermal concretions, informally called “Ace chimney” (27°N00.671′/111°W24.262′), sampled in November 2018 during *Alvin* dives 4994 and 5000, yielding predominately Desulfobacterales in sulfate-reducing enrichments on mixed aromatic hydrocarbons (Supplementary Figure 1 and Supplementary Table 1). **(C)** Oil-soaked concretions on the eastern approach to Rebecca’s Roost (27°N00.686′/111°W24.379′), sampled in December 2016 during *Alvin* dive 4872, amended with mixed n-alkanes (C_6_–C_12_) and crude oil, and yielding enrichments of alkane-oxidizing sulfate-reducing bacteria (Desulfobacterales) at 30 and 55°C (Renxing et al., 2021). Photos by *Alvin* group, Woods Hole Oceanographic Institution.

Marine hydrocarbon seeps are common features of marine sediments worldwide that release complex and variable cocktails of hydrocarbon compounds depending on interactive physical, chemical, and microbial processes (e.g., Ruff et al., 2015; Levin et al., 2016). The origin and the microbial degradation of hydrocarbons have been studied extensively in the sedimented hydrothermal system of Guaymas Basin, a young spreading center in the central Gulf of California where hot basaltic sills intrude laterally into organic-rich seafloor sediments (Einsele et al., 1980). Within the thermal aureole, petroleum hydrocarbons are produced by pyrolysis of buried organic matter under high pressure and temperature (e.g., Kawka and Simoneit, 1994), and then migrate along hydrothermal flow paths to emerge at the seafloor in hydrocarbon-soaked sediment patches and mineral concretions (Figure 1). Gas chromatography Mass Spectrometry (GCMS) analyses show that the hydrothermal oils released in Guaymas Basin show compositional similarities to reservoir crude oil (Didyk and Simoneit, 1989), containing *n*-C_12_ to *n*-C_38_ alkanes, C_27–36_ hopanes, steranes and diasteranes, and aromatic hydrocarbons [including various naphthalenes, methylnaphthalenes, phenanthrenes (including alkylated), fluoranthrene, and pyrene]. Lower concentrations of n-alkanes and aliphatic hydrocarbons and lower (<1) *n*-C_17_/pristine and *n*-C_18_/phytane ratios closer to the sediment surface are consistent with microbial biodegradation of those compounds (Bazylinski et al., 1988). Recent chemometric analysis of Guaymas Basin sediments has identified >5,000 hydrocarbon compounds that display systematic temperature-dependent trends, and complex spatial distribution patterns strongly affected by migration, biodegradation, water washing, and pyrolytic activity (Dalzell et al., 2021). These hydrocarbon-rich hydrothermal sediments provide excellent opportunities for investigating sedimentary microbial hydrocarbon biodegradation because seep sediment microbiota have been exposed to long-term selection pressure by hydrocarbon exposure.

Hydrocarbons differ in their susceptibility to microbial attack, with the most easily utilized compounds being linear alkanes followed by increasingly recalcitrant branched alkanes, small monoaromatic hydrocarbons such as benzene, toluene, ethylbenzene, and xylenes (BTEX), cyclic alkanes, and polyaromatic hydrocarbons (PAH) (Das and Chandran, 2011). Hydrocarbons are initially activated under aerobic conditions by addition of either one or both atoms of diatomic oxygen by monooxygenase and dioxygenase enzymes, after which the molecule is usually amenable to further degradation (Prince and Walters, 2007). Complex branching hinders initial oxidation and subsequent catabolism, likely since steric hindrance prevents access of hydrocarbon-oxidizing enzymes. Typically, the most readily-degraded compounds are the normal alkanes larger than hexane as well as benzene and substituted monoaromatics, followed by smaller unbranched and branched alkanes, monocycloalkanes, and at last polycyclic aromatics. Since less complex structures and molecules are more accessible and are preferentially attacked, the biodegradation stage of a particular hydrocarbon mixture can be assessed by comparing ratios of specific compounds and abundances of molecules with different structures (Prince and Walters, 2007).

The chemical inertness of hydrocarbons poses an energetic and mechanistic challenge for microbial metabolism because of the high energy requirements necessary for cleaving the apolar C-H bond (Rabus et al., 2016). Under oxic conditions, O_2_-dependent oxygenase-catalyzed reactions add one or both atoms of diatomic oxygen using monooxygenase or dioxygenase enzymes, respectively. The initial C-H activation is essential for channeling hydrocarbons as substrates to catabolic routes and for making them amenable to further degradation (Prince and Walters, 2007). Under anaerobic conditions, there are a variety of intriguing biochemical reactions that can include Adenosin triphosphate (ATP)-dependent and ATP-independent mechanisms which can combine anaerobic hydrocarbon degradation with nitrate, sulfate or iron reduction, fermentation or syntrophic growth (Rabus et al., 2016). O_2_-independent hydrocarbon degradation often involves enzymes (Moco-dependent homodimeric metalloenzymes) that resemble nitrate reductases in utilizing molybdenum as a co-factor (Rabus et al., 2016).

Recalcitrance toward microbial degradation amplifies the ecosystem-level effects of PAHs, as they accumulate within the food web. Due to their carcinogenic and mutagenic properties they can have significant toxicological effects on benthic organisms at concentrations of ≤1 mg-PAH/kg dry sediment (e.g., Long et al., 1995). PAHs can affect growth, maturity, and fecundity of metazoans (Bridges et al., 1994), impact immune systems (Tahir et al., 1993), and alter the structure of entire benthic communities (Nance, 1991). In humans, gut microbiota can metabolize persistent PAHs but produce toxic byproducts that affect the composition and metabolic activity of the gut microbiome (Claus et al., 2016). The cascading health impacts of hydrocarbon contamination lend particular urgency to microbial hydrocarbon degradation and bioremediation research.

## Cultivation Surveys of Bacterial and Archaeal Hydrocarbon Degraders

The composition and activity of the hydrocarbon-degrading microbial community at a seep site or oil spill location depends on the availability of suitable electron acceptors (e.g., molecular oxygen, nitrate, sulfate, ferric iron, or carbon dioxide), nutrients (nitrogen, phosphorus, and trace metals), and the particular spectrum of aliphatic and aromatic hydrocarbons. Since sulfate concentrations in seawater and surficial benthic sediments are two or three orders of magnitude higher than nitrate or oxygen concentrations, sulfate-reducing bacteria are naturally ubiquitous in marine hydrocarbon seeps, and have evolved numerous lineages that oxidize aliphatic and aromatic hydrocarbons, either as specialists in utilizing specific carbon sources or as generalists who can utilize a wider substrate spectrum; collectively they are now perhaps the best-studied group of anaerobic hydrocarbon oxidizers (Davidova et al., 2018; Teske, 2019; Kleindienst and Knittel, 2020).

Model organisms for anaerobic hydrocarbon degradation have been obtained from the hydrothermal sediments of Guaymas Basin, a sedimented spreading center in the Gulf of California where sedimented organic matter is transformed to petroleum compounds under high temperature and pressure, resulting in hot gas and petroleum seepage with hydrothermal characteristics (Teske et al., 2014; Teske, 2020). The distinct microbial communities of Guaymas Basin (Teske et al., 2002) include naturally enriched hydrocarbon-degrading bacteria (Kleindienst et al., 2012) and archaea (Dombrowski et al., 2017, 2018). Their ubiquitous presence and activity in all but the hottest sediments of Guaymas Basin is revealed not only by sequencing surveys but also by selective ^13^C-carbon enrichment in light alkanes, caused by preferential microbial oxidation of ^12^C-carbon compounds (Dowell et al., 2016; Song et al., 2021).

Microbial habitat for aerobic hydrocarbon degradation is confined to surficial sediment, since oxygen penetrates only the upper millimeter of Guaymas Basin sediments, with occasional small pockets of oxygenated seawater reaching deeper in hydrothermal spots with active circulation (Winkel et al., 2014; Teske et al., 2016). So far, the spectrum of aerobic hydrocarbon-degrading bacterial isolates from Guaymas Basin is limited to phenanthrene-degrading aerobic isolates of *Cycloclasticus*, *Halomonas*, *Thalassospira*, and *Lutibacterium* within the Gammaproteobacteria (Gutierrez et al., 2015), aerobic isolates with a preference for aromatic organic acids (Goetz and Jannasch, 1993), and aerobic hexadecane- and naphthalene-utilizing bacteria (Bazylinski et al., 1989).

In contrast, anaerobic, sulfate-replete sediments are abundant in Guaymas Basin, and provide ample habitat for diverse sulfate-reducing hydrocarbon degraders with different substrate and temperature preferences. The spectrum of sulfate-reducing isolates and enrichments from this frequently sampled model site includes thermophiles, such as the decane-oxidizing bacterium *Desulfothermus naphthae* strain TD3 (Rüter et al., 1994), a propane-oxidizing bacterial enrichment dominated by *Desulfotomaculum* strain Propane60-GuB (Kniemeyer et al., 2007), and the thermophilic, hydrogenotrophic sulfate-reducing bacterium *Candidatus* Desulfofervidus auxilii that grows in syntrophic association with methane- and butane-oxidizing anaerobic archaea (Krukenberg et al., 2016). In stable isotope labeling experiments using butane and dodecane as carbon sources, dominant phylotypes were affiliated with the genera *Desulfosarcina*, *Desulfococcus*, and *Desulfonema* within the *Desulfobacteraceae* (Kleindienst et al., 2014), for example the mesophilic sulfate-reducing n-butane and propane oxidizer BuS5 from Guaymas Basin (Kniemeyer et al., 2007). Enrichments on benzene from Guaymas Basin sediments yielded sequences of the *Desulfobacteraceae* and of the *Desulfatiglans* lineage (Phelps et al., 1998), represented in pure culture by the ethylbenzene-oxidizing strain EbS7 (Kniemeyer et al., 2003).

In addition to Guaymas Basin, cold seep sediments of the Gulf of Mexico have been studied extensively for anaerobic hydrocarbon degradation (briefly reviewed in Teske, 2019), and turned out to harbor sulfate-reducing populations that oxidize alkanes and polycyclic aromatic compounds. For example, anaerobic degradation of hexadecane and phenanthrene coupled with sulfate reduction was studied and quantified using enriched consortia isolated from Gulf of Mexico seafloor sediments (Shin et al., 2019). The observed stoichiometric ratios for hexadecane and phenanthrene degradation (moles of carbon source degraded per mole of SO_4_^2–^ reduced) matched the theoretical stoichiometric degradation ratios for hexadecane (12:1) and phenanthrene (8:1) when coupled to sulfate reduction (So and Young, 2001; Shin et al., 2019). Phenanthrene carboxylic acid was detected indicating active carboxylation, while metagenome-assembled genomes (MAGs) revealed that phenanthrene degradation is likely mediated by novel genera or families of sulfate-reducing bacteria along with their fermentative syntrophic partners (Shin et al., 2019).

Methane and short-chain alkanes are anaerobically activated by methyl- and alkyl-Coenzyme M reductase and stoichiometrically oxidized to CO_2_ by archaeal enrichments and isolates from Guaymas Basin, such as the thermophilic methane-oxidizing (ANME-1) archaeal lineage (Holler et al., 2011; Wegener et al., 2015), the thermophilic propane- and butane-oxidizer *Candidatus Syntrophoarcheum* sp. (Laso-Peìrez et al., 2016), and the thermophilic ethane oxidizer *Candidatus* Ethanoperedens thermophilum (Hahn et al., 2020); the latter is a sister taxon to the mesophilic ethane oxidizer *Candidatus* Argoarchaeum from cold seeps in the Gulf of Mexico (Chen et al., 2019). The thermophilic alkane-oxidizing archaea grow in syntrophic association with the thermophilic sulfate reducer *Candidatus* Desulfofervidus (Krukenberg et al., 2016).

In addition to terminal hydrocarbon oxidation to CO_2_, anaerobic microbial degradation of aliphatic and aromatic hydrocarbons can be coupled to methane production (Siddique et al., 2007, 2011). There are at least two different—syntrophic and non-syntrophic—modes of mesophilic long-chain alkane degradation to methane: syntrophic hexadecane degradation to acetate and hydrogen is performed by the deltaproteobacterial genus *Syntrophus*, coupled to acetoclastic and hydrogenotrophic methanogenesis by *Methanosaeta*- and *Methanoculleus*-related strains (Zengler et al., 1999), and syntrophic degradation of long-chain alkanes (C_28_ to C_50_ paraffins) is performed by the deltaproteobacterial genus *Smithella* associated with acetoclastic and hydrogenotrophic methanogens (Tan et al., 2014; Wawrik et al., 2016). Non-syntrophic methanogenic alkane degradation of long-chain alkanes might be catalyzed by yet-uncultured archaea such as *Candidatus* Methanoliparia that possess both methyl- and alkyl-coenzyme M reductases (nickel-containing metalloenzymes) and produce methane by disproportionating acetate using methyl-coenzyme M reductase; the acetate is obtained *via* beta-oxidation of alkanes that are activated by alkyl-coenzyme M reductase (Borrel et al., 2019; Laso-Pérez et al., 2019).

## Molecular Surveys of Bacterial and Archaeal Hydrocarbon Degraders

**FIGURE 2:**
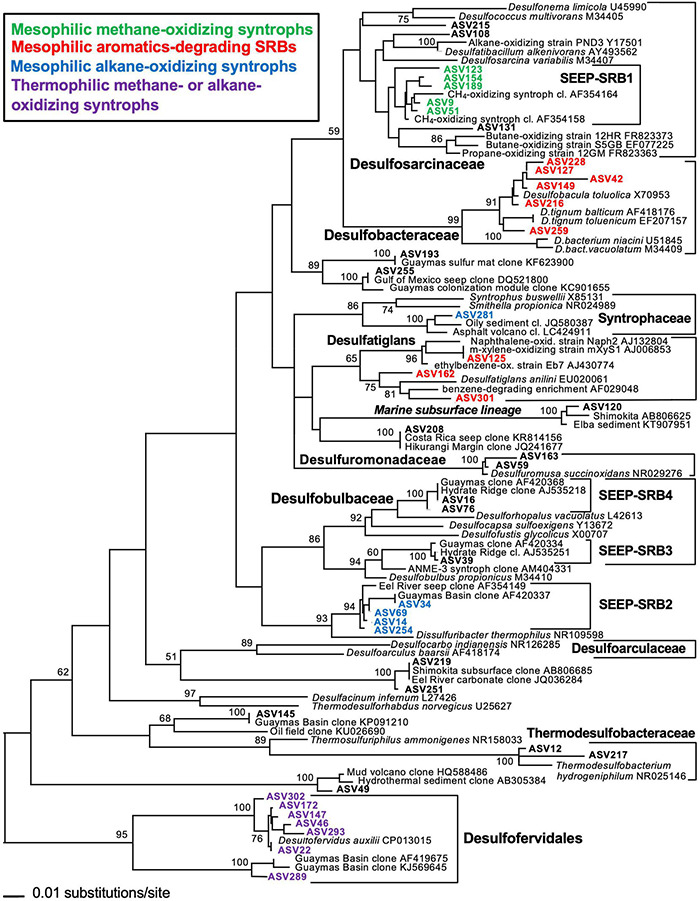
16S rRNA gene distance phylogeny of frequently occurring deltaproteobacterial ASV sequences according to Escherichia *coli* nucleotide positions 515–926 using forward primer 515F-Y and reverse primer 926R (Ramírez et al., 2021) in hydrothermal sediments of Guaymas Basin, southern axial valley. The branching pattern was checked by 1000 NJ Bootstrap iterations. The tree was rooted with the *Desulfofervidales* as outgroup. Modified by inferred substrate usage from Ramírez et al. (2021) (Supplements).

**FIGURE 3:**
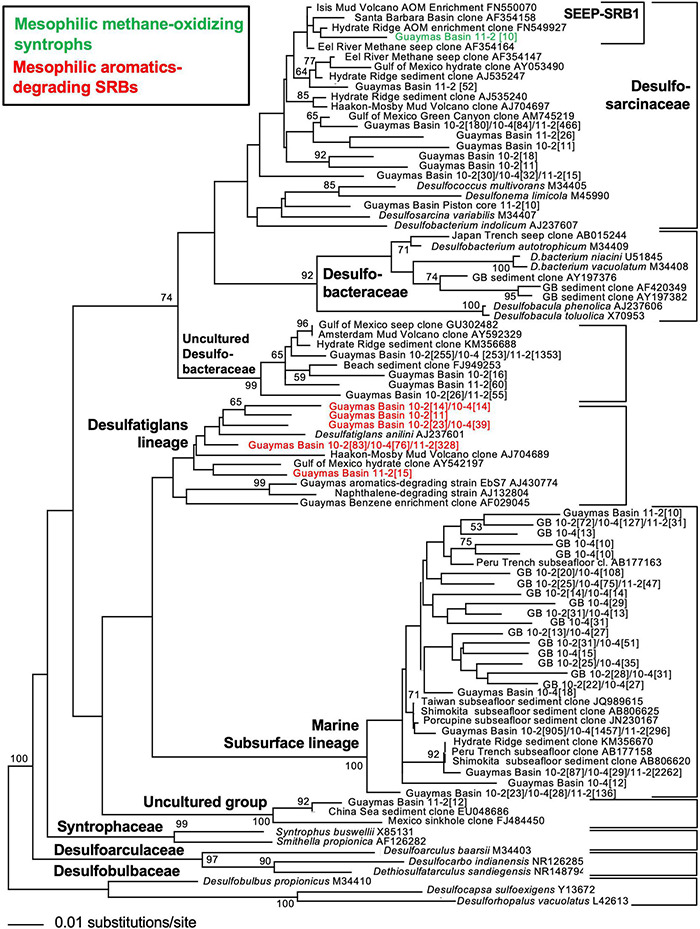
16S rRNA gene distance phylogeny of frequently occurring deltaproteobacterial ASV sequences according to *Escherichia coli* nucleotide positions 518–926 using forward primer 518F, and three versions of reverse primer 926R (Teske et al., 2019) in cold shallow subsurface sediments of Guaymas Basin, northwest of the axial valley. The taxon labels refer to number of sequences recovered from piston core 10 and 11 sediment subsections (10-2 = 1.24–1.29 mbsf; 10-4 = 3.73–3.78 mbsf; 11-2 = 1.15–1.20 mbsf). The branching pattern was checked by 1000 NJ Bootstrap iterations. Modified by inferred substrate usage, from Teske et al. (2019) (supplements).

Consistent with the phylogenetic placement of these largely deltaproteobacterial and methanomicrobial isolates and enrichments, global marker gene comparisons of hydrocarbon seep and non-seep deep-sea benthic communities have shown that members of the Deltaproteobacteria and Methanomicrobia are adapted to hydrocarbon seepage and dominate these habitats (Lloyd et al., 2010; Ruff et al., 2015). Using two case studies (Teske et al., 2019; Ramírez et al., 2021), we highlight the diversity of seep-adapted Deltaproteobacteria and Methanomicrobia by contrasting the deltaproteobacterial communities of hydrothermal hydrocarbon seep sediments in Guaymas Basin with nearby cold seafloor sediments. Small subunit ribosomal RNA (16S rRNA) gene amplicon surveys covered several types of warm and hot sediments in the hydrothermally active zone of Guaymas Basin (Ramírez et al., 2021) that were collected by push coring with the submersible *Alvin*. In contrast, off-axis shallow subsurface sediments (ca. 2–5 mbsf) were collected by piston coring outside the hydrothermal spreading center, from the northwestern flanking region of Guaymas Basin (Teske et al., 2019). Phylogenetic trees of deltaproteobacterial 16S rRNA gene sequences, obtained with near-identical primers that amplify the hypervariable V4-V5 region (Teske et al., 2019; Ramírez et al., 2021) allowed a direct comparison of hydrocarbon-degrading lineages and isolates from hot sediments of the hydrothermal spreading center and from off-axis sediments (Figures 2, 3). The hydrothermal on-axis as well as the cold off-axis survey recovered members of the *Desulfobacteraceae*, a physiologically diverse family of sulfate reducers that completely oxidize low-molecular-weight substrates such as acetate, Low molecular weight (LMW)-organic acids, alkanes, and aromatics (Küver, 2014; Teske, 2019); this group is commonly the dominant sulfate-reducing group in marine sediments (Robador et al., 2016). However, members of the SEEP-SRB1 lineage, sulfate-reducing syntrophs of methane-oxidizing archaea at cool and temperate conditions (Knittel et al., 2003; Schreiber et al., 2010) were mostly found at the hydrothermally active methane-rich sites, and were barely detectable in the cold sediments. Members of the family-level *Desulfatiglans* lineage, containing isolates that oxidize substituted mono- and polycyclic aromatics (summarized in Teske et al., 2019), were found in both hydrothermal and cold sediments (Figures 2, 3). *Desulfatiglans* phylotypes were detected as a dominant lineage even in surficial Guaymas Basin sediments that were sliced and analyzed in 2 mm intervals (Engelen et al., 2021). In addition, genome analyses indicate that Desulfatiglans-affiliated bacteria may play a role in reductive dehalogenation in marine subsurface sediments (Jochum et al., 2018).

The on- and off-axis sites share a lineage of predominantly deep subsurface clones, often annotated as members of the family Desulfoarculaceae (Davidova et al., 2016) in GenBank entries and phylogenetic analyses, but without significant bootstrap support for any phylogenetic relationship to cultured Desulfoarculaceae (Teske et al., 2019). While it is represented only by a single Amplicon sequence variant (ASV) in the hydrothermal on-axis sites (ASV 120; Figure 2), this uncultured lineage (termed “*marine subsurface lineage*” to distinguish it from proper Desulfoarculaceae) is the most frequently detected deltaproteobacterial lineage in off-axis subsurface sediments (Figure 3). It has been recovered frequently from deep-drilling surveys offshore Taiwan, the Shimokita peninsula of Japan, the Porcupine bight abyssal seafloor, and the Peru Margin and Peru Trench. A second lineage of independently branching deep subsurface clones is also annotated as a member of the Desulfoarculaceae, and occurs in the hot sediments (ASVs 219 and 251). The physiological capabilities of these misidentified “Desulfoarculaceae” remain unknown.

Several deltaproteobacterial lineages were only detected in the hydrothermal sediments (Figure 2): The *Desulfuromonadales* encompass sulfur-reducing, iron-reducing, and fermentative bacteria that commonly thrive on acetate and other LMW organic acids (Küver et al., 2005). The *Syntrophaceae* include sulfate-reducing but also fermentative bacteria that grow in syntrophic association with H_2_/formate-utilizing bacterial partners (Küver, 2014). Uncultured lineages within the incompletely oxidizing family *Desulfobulbaceae* include the seep-associated lineages SEEP-SRB3 and SEEP-SRB4, and the mesophilic, sulfate-reducing, potentially alkane-oxidizing SEEP-SRB2 lineage (Kleindienst et al., 2012; Krukenberg et al., 2018). These lineages, originally described from cold seep sites (Knittel et al., 2003), occur predominantly in temperate (not hot) hydrothermal sediments (Ramírez et al., 2021). Two lineages, the *Thermodesulfobacteraceae* and the *Desulfofervidales*, are unequivocally linked to sulfate reduction at high temperatures. The *Thermodesulfobacteraceae* have temperature optima of 65–75°C and can grow lithoautotrophically with CO_2_/H_2_ or heterotrophically with LMW organic acids (Jeanthon et al., 2002). The *Desulfofervidales*, a deeply branching 16S rRNA lineage (McKay et al., 2016) that affiliate with the Deltaproteobacteria based on complete genome analyses (Waite et al., 2020), have a growth optimum of 50–60°C and form syntrophic consortia with methane and short-chain alkane-oxidizing thermophilic archaea (Laso-Peìrez et al., 2016; Hahn et al., 2020), but they can also thrive as free-living hydrogenotrophs (Krukenberg et al., 2016). Both thermophilic lineages, the *Thermodesulfobacteraceae* and *Desulfofervidales*, are found in increasing relative proportion in deeper and hotter sediments (Ramírez et al., 2021). In general, members of the *Desulfofervidales* belong to the most frequently found lineages in Guaymas Basin and appear consistently in these hydrothermal sediments (Dowell et al., 2016; McKay et al., 2016; Engelen et al., 2021).

To summarize, hydrothermal seep sediments of Guaymas Basin harbor a high diversity of hydrocarbon-degrading Deltaproteobacteria, which participate in hydrocarbon degradation either individually or in syntrophic association with specialized archaea, whereas cold sediments from the same region contain a considerably more restricted range of Deltaproteobacteria. However, pure culture isolations and sequencing surveys cannot reveal whether these sulfate reducers might benefit from mutualistic interactions and whether they show enhanced activity in the presence of the second major group of hydrocarbon-degrading organisms that share the same sedimentary habitat, the Fungi.

## The Potential for Deep-Sea Hydrocarbon-Degrading Fungi

In contrast to bacterial and archaeal communities, unicellular microeukaryotes in deep-sea water columns and sediments are understudied, particularly in hydrothermal seep sediment habitats (Edgcomb et al., 2002). Microeukaryotes include protists and Fungi (filamentous fungi and yeasts), which are essential components of the marine microbial food web (Azam et al., 1983; Worden et al., 2015). The “mycoloop” concept suggests Ascomycetes and (to a lesser extent) Basidiomycete fungi participate in overlooked pathways salvaging substrates and energy, and thus shape aquatic and benthic ecosystems (Kagami et al., 2007, 2014). Fungi have even been posited to provide a source of energy to deep biosphere archaea and bacteria who could utilize hydrogen produced under anaerobic conditions by fungal hydrogenosome-based metabolism (Drake et al., 2017). Deep-sea seep fungi must be able to cope with elevated hydrostatic pressure and temperature. While evidence for piezophilic fungi is limited (Peng et al., 2021), some strains can tolerate pressures up to 40 MPa, and in some cases show sporulation and mycelial growth (Lorenz and Molitoris, 1997; Raghukumar and Raghukumar, 1998; Damare et al., 2006; Singh et al., 2010; Burgaud et al., 2015). Fungi can alter membrane and cell wall proteins as well as increase saturated fatty acid and ergosterol concentrations to maintain membrane fluidity and function when exposed to higher hydrostatic pressures (Fernandes et al., 2004; Iwahashi et al., 2005; Simonato et al., 2006). At hydrothermal sites like Guaymas Basin, sediment temperatures can quickly reach the upper known limit for fungi (62°C; Maheshwari et al., 2000), however, fungi appear to cope with *in situ* conditions in subsurface sediments where they have been investigated. For example, eukaryotic gene transcripts, including fungal gene transcripts, from subsurface Peru Margin and Canterbury Basin sediments (down to 159 and 350 mbsf, respectively; Orsi et al., 2013; Pachiadaki et al., 2016; Orsi, 2018) were assigned to cell growth and division, recycling of organic matter, cell-cell competition, and synthesis of antimicrobial secondary metabolites. Fungi secrete exoenzymes that break complex refractory carbohydrates in marine sediments (Orsi, 2018), and thus fungi can likely survive in deep-sea sediments and in nutrient-poor extreme environments (e.g., the lower oceanic crust) on refractory polysaccharides including peptidoglycan (Lomstein et al., 2009, 2012; Quemener et al., 2020), necromass of their microbial neighbors, and perhaps by activating carbon-starvation stress responses that include autolysis and utilization of polysaccharides from their own cell wall for survival (García-Lepe et al., 1997; Emri et al., 2005; Kim et al., 2011; Quemener et al., 2020).

Interestingly, fungi are thought to have a relatively high tolerance to hydrocarbons (Al-Nasrawi, 2012), and over 100 genera (Prince, 2005) are known to play important roles in the biodegradation of hydrocarbons in soils and sediments (e.g., Walker and Colwell, 1975; Hestbjerg et al., 2003; Šašek et al., 2003; Wiesche et al., 2003; Gesinde et al., 2008; Husaini et al., 2008; Obire and Anyanwu, 2009). Filamentous fungi such as *Cladosporium* (isolated from Guaymas Basin, Table 1) and *Aspergillus* are among those known to participate in aliphatic hydrocarbon degradation, and the genera *Cunninghamella, Penicillium, Fusarium*, *Mucor*, and *Aspergillus* are among those known to take part in degradation of aromatic hydrocarbons (Passarini et al., 2011; Al-Nasrawi, 2012 and references therein; Steliga, 2012). Culture-based studies have shown that the addition of different fungi increases the microbial degradation of aromatic hydrocarbons, such as benzene, toluene, ethylbenzene and xylene, and PAHs (i.e., Yadav and Reddy, 1993; Zheng and Obbard, 2001; Wattiau, 2002; Šašek et al., 2003; Wiesche et al., 2003; Steliga, 2008). While most filamentous fungi investigated to date are unable to fully mineralize aromatic hydrocarbons, fungi play a critical role in facilitating the degradation of more recalcitrant hydrocarbons in the environment by secreting extracellular enzymes that transform these compounds into intermediates that are increasingly susceptible to bacterial decomposition (Steliga, 2012).

**TABLE 1:** Sample site key for published fungal isolates from Guaymas Basin (Keeler et al., 2021; sampling key not reported therein).

Sample	Location	*In situ* temp.	Time of sampling	Water depth	Sediment depth	Isolates obtained	References for sampling site
*El Puma* piston core P03_4: cold seafloor sediment	Guaymas Basin NW flanking region 27°37.676/−111°52.574	3–4°C	October 17, 2014	1,611 m	276–281 cm	Redundant isolates	Teske et al., 2019; Ramírez et al., 2020
*El Puma* piston core P05_3: cold seep sediment	Sonora Margin/Guaymas Basin transition 27°38.765/−111°38.909	3–4°C	October 19, 2014	1,739 m	202–207 cm	GB_1 *Cladosporium* sp.; GB_10 *Xylaria feejeensis*	Parallel core P0_6: Teske et al., 2019; Ramírez et al., 2020
*El Puma* piston core P08_3: cold seafloor sediment	Sonora Margin slope 27°N40.342/−111°24.121	3–4°C	October 20, 2014	995 m	179–184 cm	GB_7 *Torulaspora delbrueckii*	This study
*El Puma* piston core P10_4: cold seafloor sediment	Guaymas Basin NW flanking region 27°30.519/−111°42.172	3–4°C	October 21, 2014	1,731 m	353–358 cm	GB_11 *Ramularia eucalypti*	Teske et al., 2019; Ramírez et al., 2020
*Alvin* push core 4862-4: hydrothermal sediment	Southern Guaymas Basin Mat Mound Massif	Ca. 40°C	December 13, 2016	2,000 m	26–28 cm	GB_5 *Engyodontium album*; GB_9 *Rhodotorula mucilaginosa*	Teske et al., 2021b
*Alvin* dive 4864 sample: hydrothermal silicate crusts	Off-axis Ringvent hydrothermal area 27°30.360/−111°40.870 27°30.380/−111°40.890 [ORP and Mound1 site]	Ca. 3–4°C	December 15, 2016	1,720 m	Seafloor	GB_8 *Cadophora* sp.	Escorcia-Ocampo et al., 2018; Teske et al., 2019
*Alvin* push core 4867-14, cold seep sediment	Off-axis Octopus Mound cold seep, Active site 27°28.150/−111°28.400	3°C	December 18, 2016	1,850 m	30–33 cm	GB_4 and GB_6, *Aureobasidium pullulans*; GB_12 *Dioszegia xingshanensis*	Teske et al., 2021b
*Alvin* push core 4868-12: hydrothermal sediment	Southern Guaymas Basin Mat Mound Massif 27°00.430/−111°24.520	Ca. 50°C at 20 cm	December 19, 2016	2,000 m	0–16 cm	Redundant isolates	Engelen et al., 2021;Teske et al., 2021b
*Alvin* push core 4871-10: hydrothermal sediment	Southern Guaymas Basin, Northern Towers area 27°02.680/−111°23.080	Ca. 60–70°C at 20 cm	December 23, 2016	1,995 m	Seafloor	GB_2 *Penicillium chrysogenum*; GB_3 *Cadophora malorum*	Teske et al., 2021b
*Alvin* push core 4872-13: hydrothermal sediment	Southern Guaymas Basin, Cathedral Hill area 27°00.680/−111°24.270	Ca. 40–50°C	December 24, 2016	2,000 m	19.5–52 cm	Redundant isolates	This study
*Alvin* dive 4872: hydrocarbon-rich mineral aggregate	Southern Guaymas Basin, Cathedral Hill area 27°00.700/−111°24.280	Ca. 3–50°C	December 24, 2016	2,000 m	Seafloor	Redundant isolates	This study

*This table includes strains GB_9 Rhodotorula mucilaginosa, GB_4 and GB_6 Aureobasidium pullulans, GB_1 Cladosporium sp., and GB_8 Cadophora malorum that are used for long-term incubation experiments on polyaromatic hydrocarbons. Additional isolates that were not selected to represent the final 12 non-redundant isolates (Keeler et al., 2021) are listed as “redundant isolates”.*

Fungal communities utilize diverse enzymatic mechanisms to transform hydrocarbon structures. Saturated hydrocarbons (e.g., alkanes) are oxidized using cytochrome P450 monoxygenases, while unsaturated alkenes, alkynes, and alicyclics, are more recalcitrant, and fungi are considered to be able to partially oxidize them (Prenafeta-Boldú et al., 2018). Low and high-molecular weight PAHs can be degraded using a combination of enzymatic pathways including P450 monoxygenases as well as extracellular peroxidases and laccases produced by lignin-degrading fungi that can oxidize a broad range of aromatic hydrocarbons (Prenafeta-Boldú et al., 2018). Interestingly, fungal biodegradation often results in partially degraded hydrocarbon compounds that do not support further fungal growth, and might be more toxic than the parent substrates (Prenafeta-Boldú et al., 2018). Therefore, syntrophic growth of fungi with bacteria or archaea that can utilize the products of fungal hydrocarbon metabolism, or co-cultures that allow for sequential utilization of hydrocarbon compounds and degradation products within the same habitat, may be a win-win strategy for all microbiota involved in hydrocarbon degradation.

## Fungal Biosurfactants and Hydrocarbon Degradation

The poor bioavailability of hydrocarbon constituent components is considered a major rate limiting factor in the hydrocarbon remediation process (Das et al., 2014). Biosurfactants act as surface-active amphiphilic compounds with a hydrophobic and hydrophilic moiety interacting with phase boundaries in a heterogeneous system to solubilize organic compounds (Sen et al., 2017). Thus, in the presence of biosurfactants, the chemical inertness of hydrocarbon contaminants can be reduced, and microbial degradation can occur through improved solubilization (Banat et al., 2014). Surfactants are used to enhance bioremediation of accidental hydrocarbon releases, and in oil recovery. In contrast to chemical surfactants such as carboxylates, sulfonates, sulfates (Damare et al., 2012), biosurfactants have advantages such as lower toxicity and higher biodegradability (Shekhar et al., 2015). While bacterial biosurfactants are common, often produced by members of the genera *Pseudomonas, Acinetobacter*, and *Bacillus* (Soberón-Chávez and Maier, 2011), the importance of production of biosurfactants by yeasts and filamentous fungi is increasingly recognized; of total available biosurfactants 12% are from ascomycetes and 7% from basidiomycetes. Fungal biosurfactants are unique and include sophorolipids, mannosylerythritol lipids, cellobiose lipids, xylolipids, lipid polyols, and hydrophobins, all known to have a wide range of applications including in environmental remediation (da Silva et al., 2021 and references therein).

Known producers are affiliated to yeasts of the genera *Candida*, *Pseudozyma*, or *Rhodotorula* (Diniz Rufino et al., 2014; Sajna et al., 2015; Sen et al., 2017) and to filamentous fungi of the genera *Cunninghamella*, *Fusarium*, *Phoma*, *Cladophialophora*, *Exophiala*, *Aspergillus*, and *Penicillium* (Silva et al., 2014; Lima et al., 2016). Biosurfactants also remain active under harsh conditions of temperature, pH, and salinity. As an example, biosurfactants produced by a yeast affiliated to the genus *Rhodotorula* exhibit high stability over a wide range of temperature (at 120°C for 30–120 min), salinity (2–10% NaCl), and pH (2–10) (Sen et al., 2017). Bacterial isolates from hydrocarbon-contaminated samples, such as *Pseudomonas* (Patowary et al., 2017) and *Halomonas* (Gutierrez et al., 2013) also synthesize biosurfactants while growing on crude oil components as carbon sources, thereby improving hydrocarbon degradation further by facilitating microbial access. Considering the plethora of microorganisms that produce biosurfactants (Cameotra and Makkar, 2010), and the existence of unique fungal biosurfactants, it is possible some Fungi play a critical role in facilitating the bio-availability of hydrocarbons to other microbial populations (i.e., to other Fungi or Bacteria) and by synthesizing biosurfactants.

## Fungal Isolations and Fungal Diversity in Guaymas Basin

An in-depth investigation of Guaymas Fungi, their diversity, hydrocarbon-degrading capabilities, and abilities to enhance bacterial hydrocarbon degradation—for example, in a consortium of fungal and bacterial hydrocarbon degraders—is currently being conducted in the Edgcomb lab. The fungal project started with a sequencing survey that detected diverse fungal communities in Guaymas hydrothermal sediments using fungal ITS and 18S rRNA gene amplicons (Ramírez et al., 2021). Microeukaryotic signatures included previously described hydrocarbonoclastic fungal taxa (e.g., *Aureobasidium* sp., *Penicillium* sp., *Cladosporium* sp.), as well as basal fungi (e.g., chytrids) known to be degraders of recalcitrant carbon sources (Hassett et al., 2020; Ramírez et al., 2021). Fungal signatures in surficial sediment layers (0–30 cmbsf) of Guaymas Basin were dominated by chytrids (Ramírez et al., 2021); these zoosporic fungi are included among “fungal dark matter” due to unresolved biological questions regarding their life cycle and evolution, as well as their roles in the marine environment (Grossart et al., 2016; Naranjo-Ortiz and Gabaldo, 2019; Laundon and Cunliffe, 2021).

**FIGURE 4:**
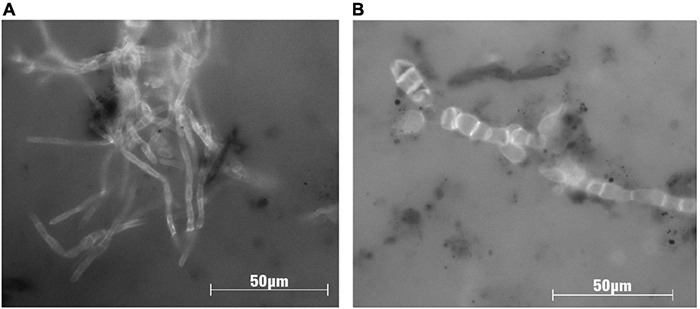
Microphotographs of Guaymas Basin fungal strains imaged under fluorescence after calcofluor staining, which highlights the chitin cell walls of fungi. Strains were isolated from Guaymas Basin sediments (Table 1), and images were taken after 40 days of incubation with the polyaromatic substrate naphthalene (5 μg/ml) in co-culture with Desulfobacterales-dominated sulfate-reducing bacteria. Panel **(A)** shows growth of the filamentous ascomycete fungus *Cadophora malorum*, and panel **(B)** shows filamentous arrangement of cells of the yeast-like ascomycete fungus *Aureobasidium pullulans*.

In parallel to the sequencing survey, an initial cultivation survey (Keeler et al., 2021) using hydrothermal and non-hydrothermal sediment inocula from Guaymas Basin yielded 12 distinct isolates of Ascomycota and Basidiomycota, after dereplication to account for redundant isolates (Table 1, Figure 4). Ten isolates showed growth based on optical density and light microscopy during 24 h of incubation at 25°C at *in situ* pressures in stainless steel pressure vessels (High Pressure Equipment Co., Erie, PA, United States). Interestingly, our isolate of the wide-spread marine yeast *Rhodotorula*, obtained from microbial-mat covered hydrothermal sediment in the Mat Mound Massif area (Teske et al., 2021a), maintained culture cell densities over 3 weeks in liquid medium with naphthalene substituted as the sole C source under reducing, anoxic conditions (100x *in situ* naphthalene concentration provided). To evaluate the role of fungi in hydrocarbon degradation, a deeper culturing effort was needed in order to obtain the broadest possible diversity of *in situ* fungi from different sites and depths. The Edgcomb lab is collaborating with Gaetan Burgaud at the University of Brest, France, to carry out high-throughput fungal culturing efforts. This collaboration has yielded ∼200 unique fungal isolates from subsurface Guaymas sediments, and all isolates in the collection are currently being tested for production of biosurfactants.

A persistent issue is that publications generally refer to aerobic hydrocarbon degradation by fungi, whereas anaerobic degradation is attributed to bacteria. For example, hexadecane is oxidized under aerobic conditions by fungi isolated from surficial marine sediments in the Gulf of Mexico (Velez et al., 2020), whereas anaerobic degradation of hexadecane in marine sediments is performed by sulfate-reducing bacteria (So and Young, 2001; Cravo-Laureau et al., 2005; Shin et al., 2019). As a possible interaction between fungi and sulfate-reducing bacteria, fermentative anaerobic fungi could supply sulfate-reducing bacteria with H_2_ or acetate (Ivarsson et al., 2018), to provide sulfate-reducing microbial communities with a head start and to create suitably reducing conditions that allow anaerobic hexadecane degradation by sulfate-reducing bacteria. In the case of recalcitrant polyaromatics, such as the non-substituted 4-ring components pyrene and chrysene, the available evidence suggests that aerobic fungi initiate this process, whereas anaerobic degradation of polyaromatics, as far as known, is a slow-acting bacterial process. Multiple studies have shown that pyrene can be metabolized by fungi aerobically (Ravelet et al., 2000, 2001; da Silva et al., 2003). Chrysene is metabolized by some aerobic fungal strains producing napthoic acid (Hadibaratam et al., 2009), and is completely remineralized to CO_2_ by co-cultures of fungi with bacteria in soils (Boonchan et al., 2000). Yet in anaerobic marine sediments exposed to long-term hydrocarbon pollution, chrysene and pyrene are slowly degraded under anaerobic, sulfate-reducing conditions, by 25 and 13% of the original concentration, respectively, after 11 months of incubation (Rothermich et al., 2002). Controls with the sulfate-reducing inhibitor molybdate did not show any polyaromatic degradation, leading to the conclusion that specifically sulfate-reducing bacteria were the key agents of chrysene and pyrene degradation (Rothermich et al., 2002). In pyrene-amended anoxic incubations with 10–20% (v/v) inoculum of contaminated sludge, a (non-sulfate-reducing) *Klebsiella* isolate degraded pyrene *via* the intermediate phenanthrene to substituted monoaromatic compounds (Li et al., 2018).

**FIGURE 5:**
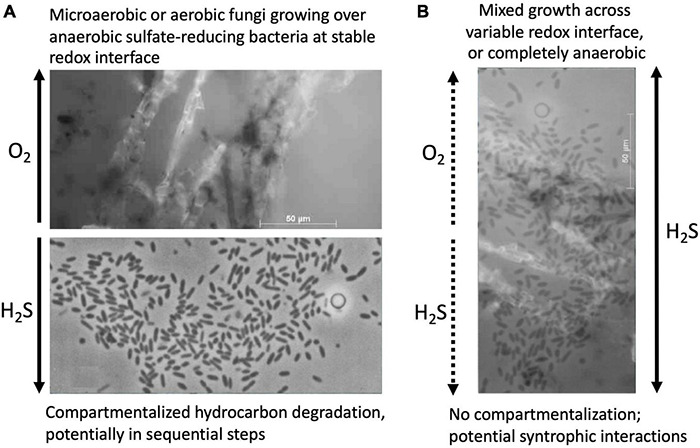
Concept sketch for potential interactions of hydrocarbon-degrading fungi and sulfate-reducing bacteria in Guaymas Basin sediments. **(A)** Spatially separated, redox-stratified niches for aerobic hydrocarbon-degrading fungi and sulfate-reducing anaerobic bacteria, implying compartmentalized hydrocarbon degradation in distinct steps. **(B)** Co-occurrence and potential syntrophic associations of hydrocarbon-degrading fungi and sulfate-reducing bacteria in sediments with fluctuating redox gradients, or fully sulfidic conditions, visualized by superimposition of both microphotographs. The fungal isolate used for this sketch is *Cladosporium*_GB1 (Table 1) grown on naphthalene as sole carbon source; the bacterial image shows *Desulfothermus naphthae* strain TD3, a thermophilic hexadecane-degrading sulfate reducer from Guaymas Basin sediments (Rüter et al., 1994).

Given the well-documented potential for anaerobic degradation of long-chain alkanes and polycyclic aromatics in anoxic marine sediments, we speculate that highly recalcitrant hydrocarbons can be metabolized in multiple redox zones in Guaymas Basin, for example by aerobic cracking of polyaromatic rings in surficial sediments (Gutierrez et al., 2015), and also in the organic-rich sediments immediately underneath that host a diverse cohort of sulfate-reducing bacteria with different substrate preferences. Since sulfate reduction rate maxima are found consistently in the upper 4 cm of Guaymas Basin sediments (Meyer et al., 2013), and oxygenated bubbles are circulating in hydrothermally active, sulfate-replete surficial sediments (Gundersen et al., 1992; Teske et al., 2016), aerobic and sulfate-reducing degradation processes could occur simultaneously, at least in close proximity to each other at the redox interface or on overlapping spatial scales in mixed communities that thrive in dynamic redox gradients (Figure 5). The active microbiota might involve aerobic as well as anaerobic fungi that share the surficial sediment habitat with similarly diverse bacteria.

Given the co-occurrence of fungi and sulfate-reducing bacteria in Guaymas Basin sediment (Ramírez et al., 2021), we are exploring the efficacy of co-cultures of fungal isolates and sulfate-reducing bacterial enrichments to degrade selected hydrocarbons. Under controlled lab conditions, fungal isolates are being used individually and in co-culture experiments with hydrocarbon-degrading sulfate-reducing bacterial enrichments (dominated by members of the Desulfobacterales, Supplementary Figure 1 and Supplementary Table 1), but supplemented with short-term oxygen spikes, comparable to the impact of hydrothermal circulation and seawater inmixing into surficial Guaymas Basin sediments. As a first step, cultures of fungi alone, bacterial enrichments alone, and bacterial-fungal co-cultures are incubated with a mixture of aliphatic and polyaromatic hydrocarbons in sulfate-reducing media (Widdel and Bak, 1992). The Guaymas sediments that were used to initiate the bacterial enrichments had been conditioned by adding benzoate (0.5 mM) and acetate (2 mM), to start sulfate-reducing activity; benzoate is a successful substrate for bacterial isolations from Guaymas Basin under aerobic (Goetz and Jannasch, 1993) and sulfate-reducing conditions (Phelps et al., 1998). Once the fungal/SRB hydrocarbon incubations were established in stoppered serum bottles and sulfide production indicated robust sulfate-reducing activity, weekly injections of oxygen in low concentrations (∼5–10% headspace concentration) were added to some replicates to imitate the biogeochemical regime that can occur in Guaymas Basin hydrothermal sediments, where oxygenated seawater percolates through surficial anaerobic and sulfide-rich sediment and introduces oxygen “bubbles” into an otherwise anaerobic and sulfidic sediment (Teske et al., 2016). Under these conditions, some Guaymas Basin fungal isolates that were originally isolated aerobically can maintain their presence in the sulfate-reducing enrichment cultures, and coexist with mixed populations of sulfate-reducing bacteria. Incubations without oxygen additions and fungal inoculum are running in parallel, to serve as controls for purely anaerobic hydrocarbon-degrading activity by sulfate-reducing bacteria. The long-term incubation experiment is currently in its eighth month; in suitable intervals subsamples are collected and analyzed for microbial community composition and hydrocarbon degradation using 16S rRNA gene itag sequencing and two-dimensional gas chromatography (Ventura et al., 2012).

## Future Directions

There is growing evidence that hydrocarbon biodegradation is an active process in Guaymas Basin sediments. Yet there are many avenues of investigation to pursue in order to obtain a more complete understanding of the roles played by diverse microorganisms in the utilization of hydrocarbons as an energy source. The recent International Ocean Discovery Program Expedition 385 (IODP X385) drilled hundreds of meters into the seafloor of Guaymas Basin at eight different sites (Supplementary Figure 2). The thickly sedimented northwestern spreading region with deeply emplaced sills is represented by sites 1545 and 1546 (Teske et al., 2018), Sites 1547 and 1548 are placed into the center and the periphery of the hydrothermal Ringvent site with a hot, shallow sill (Lizarralde et al., 2011; Soule et al., 2018; Teske et al., 2019), Site 1549 drills into the vicinity of a methane-rich cold seep, termed Central Seep (Geilert et al., 2018; Teske et al., 2021a), Site 1550 samples the northern axial trough of Guaymas Basin, Site 1551 represents the massive terrigenous sediments of the southeastern spreading region, and Site 1552 samples one of the numerous cold seep sites adjacent to the Sonora Margin (Portail et al., 2015; Teske et al., 2018). All Site reports are available at http://iodp.tamu.edu/publications/proceedings.html. These deep subsurface sediments vary significantly in their thermal, biogeochemical, and microbial cell number profiles. Comparing sites where temperatures increase steeply above shallow, hot sills, and sites with more gradual temperature gradients offer a chance to systematically examine the influences of temperature and pressure. In-depth analyses of taxonomic diversity, genome content, and cell abundances along cores from these eight sites will inform on the distribution of deep biosphere microbiota (bacteria, archaea, and microbial fungi) in this hydrothermal setting along temperature, depth and substrate gradients. Correlation analyses, analyses of genomic potential in metagenomes and MAGs, and studies of cultured fungal isolates will reveal the likely nutritional sources (e.g., microbial necromass, buried organic material) that allow bacteria, archaea, and fungi to survive in this habitat.

Periodic historical magmatic sill intrusions, which have “baked” buried sediments surrounding each sill, invariably have had a major effect on the modern pool of available nutrients remaining in sediments in close proximity to buried sills (Teske et al., 2014). Current and future research will determine how these historic events shape the modern chemical composition of those sediments and whether microbiota are able to survive on remaining organic substrates. Analysis of the mRNA pool, proteome, and secretome isolated from samples along the depths drilled at each site are necessary to inform on the active fraction of the community and on microbial strategies for coping with increasing heat and the stresses of ever-scarcer nutrients with depth. The application of recently developed molecular methods such as bioorthogonal non-canonical amino acid tagging (BONCAT), RNA-SIP, and live/dead stains applied to isolated cells are needed to confirm results from interpretations of molecular data as to the fraction of the *in situ* community that is actively contributing to nutrient cycling in the deep subsurface Guaymas biosphere (Teske et al., 2021c). In the anoxic deep biosphere, we may discover diverse fungal-bacterial syntrophic interactions, that allow the syntrophic partners to survive by adapting to different sources of carbon and/or energy, and by cooperating in accessing substrates that individual taxa cannot access alone.

Finally, studies of enrichment cultures, cultured isolates, and co-cultures can provide critical information on whether interactions between microbial taxa enhance their ability to access otherwise refractory organic carbon substrates found in Guaymas Basin sediments, including diverse hydrocarbons. Promising isolates and enrichments could possibly be engineered to remediate and clean-up oil spills with minimal ecosystem disturbance. For example, experiments that prime indigenous soil bacteria capable of hydrocarbon degradation for oil bioremediation show that horizontally transferred gene vectors encoding genes for petroleum hydrocarbon degradation persist in indigenous populations only when under selection pressure, but disappear when the hydrocarbon source is removed (French et al., 2020).

Using new molecular assays and long-term enrichments, diverse fungal-bacterial syntrophic interactions might be discovered that allow these partners to survive in the anoxic deep biosphere by adapting to different carbon and energy sources, and by cooperating in accessing substrates that individual taxa cannot access alone. In this way, it will be possible to go beyond studies of individual fungal and sulfate-reducing species and their capabilities to oxidize hydrocarbon and low-molecular weight organic compounds, and to explore the possibility that selected, fungi, fungal-bacteria co-cultures and syntrophic cultures enhance the accessibility of particular hydrocarbons through successive or simultaneous reactions.

## Data Availability Statement

The original contributions presented in the study are included in the article/Supplementary Material, further inquiries can be directed to the corresponding author.

## Author Contributions

VE led the fungal-bacterial interactions project and designed the experiments. PM performed the experiments. AT wrote the first draft of the manuscript. All authors commented and edited the manuscript in turn.

## Conflict of Interest

The authors declare that the research was conducted in the absence of any commercial or financial relationships that could be construed as a potential conflict of interest.

## Publisher’s Note

All claims expressed in this article are solely those of the authors and do not necessarily represent those of their affiliated organizations, or those of the publisher, the editors and the reviewers. Any product that may be evaluated in this article, or claim that may be made by its manufacturer, is not guaranteed or endorsed by the publisher.
