# Supplementary material for: Microbial Hydrocarbon Degradation in Guaymas Basin—Exploring the Roles and Potential Interactions of Fungi and Sulfate-Reducing Bacteria
Source: Front Microbiol. 2022 Mar 9;13:831828. doi: 10.3389/fmicb.2022.831828 (PMC8959706; doi:10.3389/fmicb.2022.831828)
Supplement: Supplementary file 1 [file Data_Sheet_1.docx]

**Supplementary Material to**

**Microbial hydrocarbon degradation in Guaymas Basin – Exploring the roles and potential interactions of fungi and sulfate-reducing bacteria**

by

**V. Edgcomb, A. Teske, P. Mara**

**Supplementary Methods**

DNA was extracted from 2ml of each of the 14 enrichments in Supplementary Table 1 in April 2020 using the 2X Lysis/CTAB extraction method (Gast et al. 2004). Partial bacterial small subunit ribosomal RNA gene sequences were amplified using Bac515F-Y and Bac926R (Parada et al. 2016). PCR products were sequenced on one run of PE Illumina MiSeq 2x300bp at MrDNA (Shallowater TX, USA). Amplicon libraries were processed for quality checking, primer removal, assembly, and annotation using Qiime 2 (Bolyen et al. 2019).

**Supplementary Table S1**. Overview on sulfate-reducing enrichments from Guaymas Basin samples in SRB medium (Widdel and Bak 1992) supplemented with different carbon sources.

**
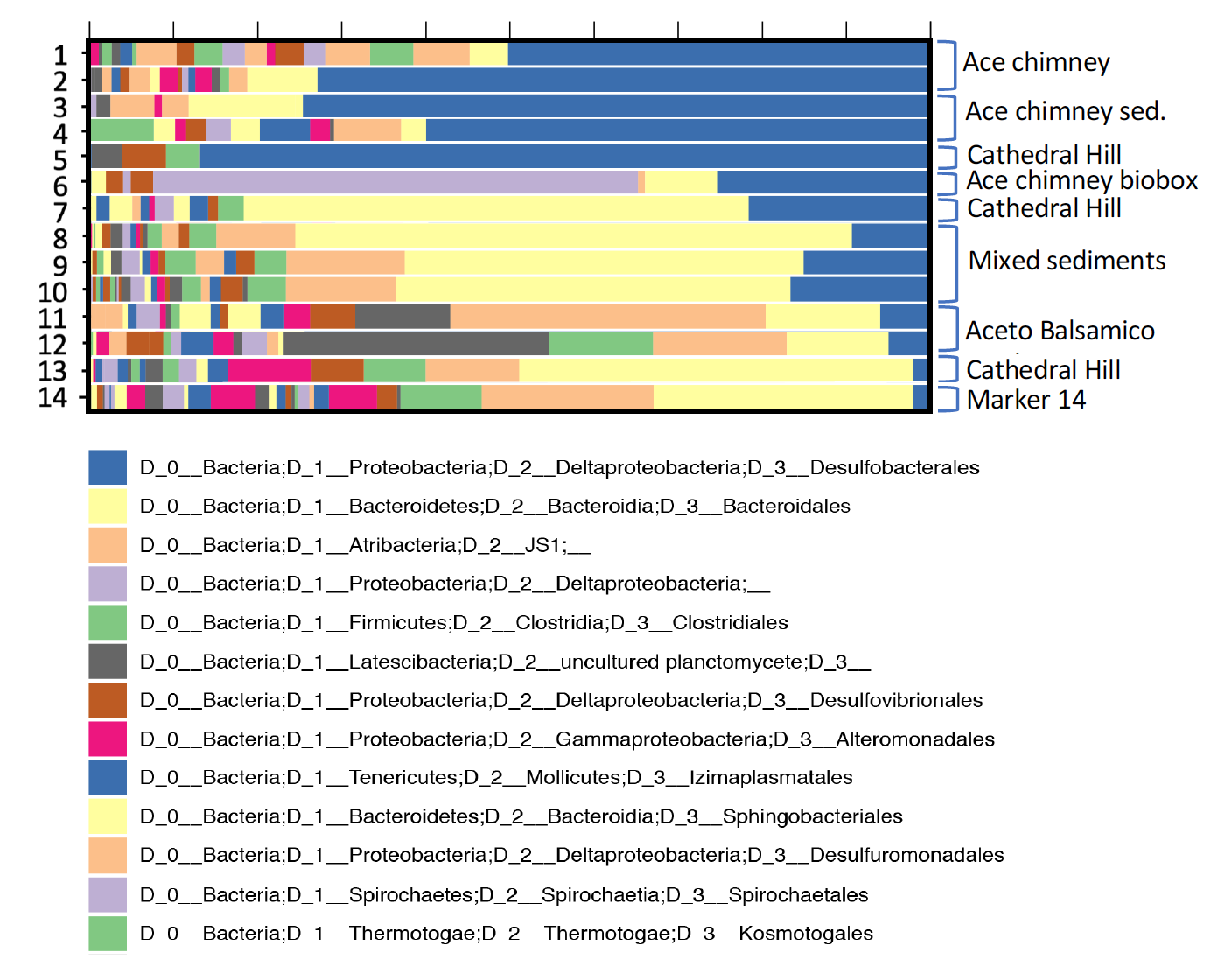
**

**Supplementary Figure S1**. Microbial community composition of sulfate-reducing enrichments from samples listed in Table S1, shown at family-level resolution. The “mixed sediment” samples (No. 8-10) were created by pooling different sediment samples (4994-31, 4995-8, 4998-15,19,21; 5000-12,19,23,24; and Ace Chimney material) and inoculating ~20 replicate 100 ml serum vials with 10 ml of the mixture, to ensure reproducible and consistent inocula. These samples were used for ongoing SRB-fungal co-culture experiments described in this paper. Sequence data deposited to GenBank BioProject accession number PRJNA642872.

.

**
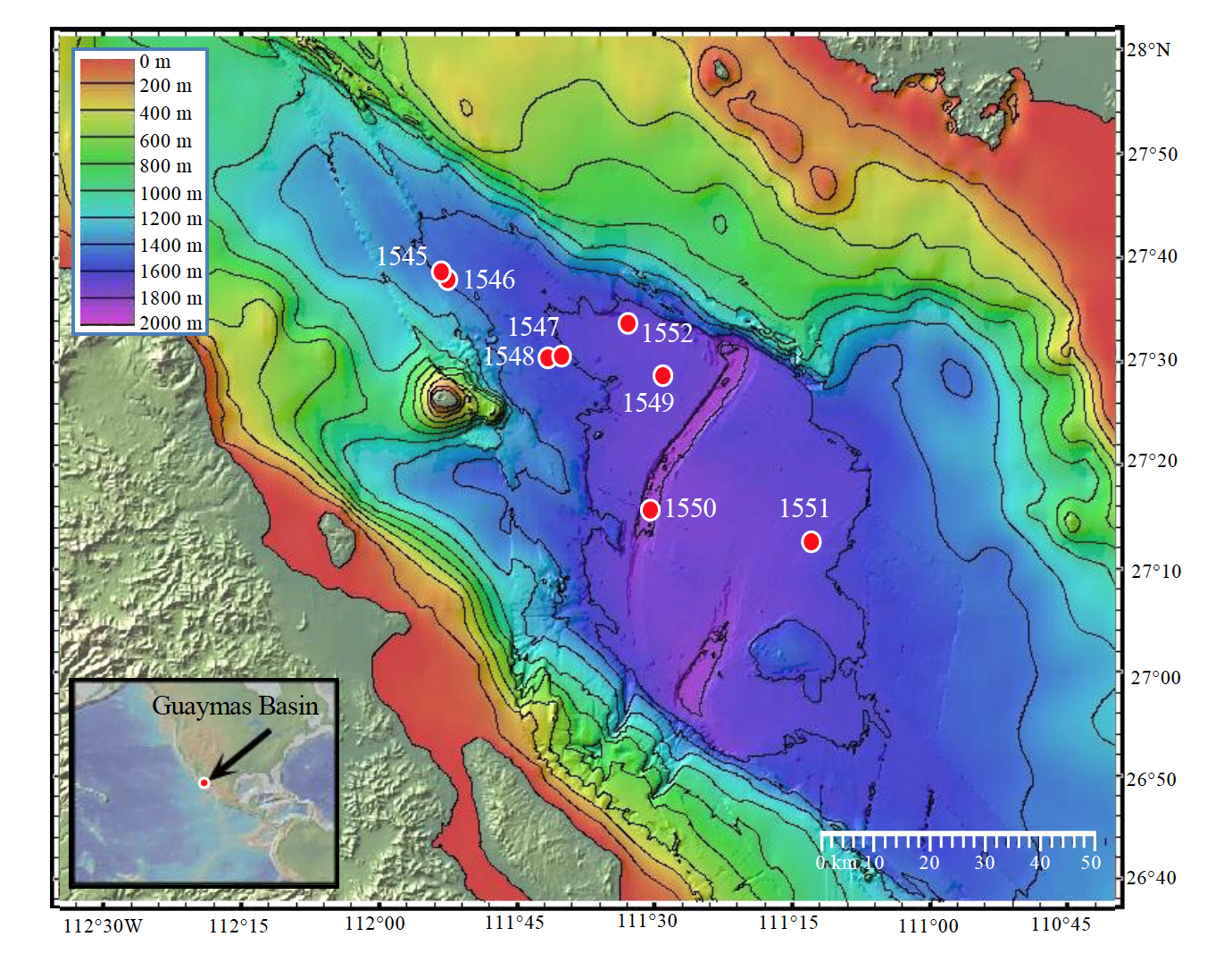
**

**Supplementary Figure S2.** Deep drilling Sites of IODP Expedition 385 in Guaymas Basin. Bathymetry of Guaymas Basin courtesy of C. Mortera, UNAM.

**Supplementary References**

Bolyen E, Rideout JR, Dillon MR, Bokulich NA, Abnet CC, Al-Ghalith GA, Alexander H, Alm EJ, Arumugam M, Asnicar F, Bai Y, Bisanz JE, Bittinger K, Brejnrod A, Brislawn CJ, Brown CT, Callahan BJ, Caraballo-Rodríguez AM, Chase J, Cope EK, Da Silva R, Diener C, Dorrestein PC, Douglas GM, Durall DM, Duvallet C, Edwardson CF, Ernst M, Estaki M, Fouquier J, Gauglitz JM, Gibbons SM, Gibson DL, Gonzalez A, Gorlick K, Guo J, Hillmann B, Holmes S, Holste H, Huttenhower C, Huttley GA, Janssen S, Jarmusch AK, Jiang L, Kaehler BD, Kang KB, Keefe CR, Keim P, Kelley ST, Knights D, Koester I, Kosciolek T, Kreps J, Langille MGI, Lee J, Ley R, Liu YX, Loftfield E, Lozupone C, Maher M, Marotz C, Martin BD, McDonald D, McIver LJ, Melnik AV, Metcalf JL, Morgan SC, Morton JT, Naimey AT, Navas-Molina JA, Nothias LF, Orchanian SB, Pearson T, Peoples SL, Petras D, Preuss ML, Pruesse E, Rasmussen LB, Rivers A, Robeson MS, Rosenthal P, Segata N, Shaffer M, Shiffer A, Sinha R, Song SJ, Spear JR, Swafford AD, Thompson LR, Torres PJ, Trinh P, Tripathi A, Turnbaugh PJ, Ul-Hasan S, van der Hooft JJJ, Vargas F, Vázquez-Baeza Y, Vogtmann E, von Hippel M, Walters W, Wan Y, Wang M, Warren J, Weber KC, Williamson CHD, Willis AD, Xu ZZ, Zaneveld JR, Zhang Y, Zhu Q, Knight R, and Caporaso JG. 2019. Reproducible, interactive, scalable and extensible microbiome data science using QIIME 2. *Nature Biotechnology* **37**: 852–857. <https://doi.org/10.1038/s41587-019-0209-9>

Gast, R.J., Dennett, M.R., Caron, D.A. 2004. Characterization of protistan assemblages in the Ross Sea, Antarctica, by denaturing gradient gel electrophoresis. *Applied and Environmental Microbiology* doi:10.1128/AEM.70.4.2028-2037.2004.

Parada, A.E., Needham, D.M., Fuhrman, J.A. 2016. Every base matters: assessing small subunit rRNA primers for marine microbiomes with mock communities, time series and global field samples. *Environ. Microbiol*. 18:1403-14, doi:10.1111/1462-2920.13023.

Widdel, F., and Bak, F. (1992). Gram-negative mesophilic sulfate-reducing bacteria. Pp. 3352-3378. In: The Prokaryotes. Edited by A. Balows, H.G. Trüper, M. Dworkin, W. Harder, and K.H. Schleifer. Second edition, Springer Verlag, New York.
